# Obstetrical Cases

**Published:** 1860-10

**Authors:** T. P. Bailey

**Affiliations:** North Santee, South Carolina


### SELECTIONS.

*Obstetrical Cases.* By T. P. Bailey, M. D., of North Santee, South Carolina.

Case I.—June 20th, 1858. Delia, a negro woman, aged about 40, the mother of several children, was taken in labor early in the afternoon. About sunset, the membranes ruptured, and she was delivered of an infant that presented in the ordinary way. In a very few minutes after, pains came on and the arm of a second child presented, which the midwife tried to extricate by traction, hoping to expedite the labor in this way, and thus wedging the arm and shoulder in the inferior strait of the pelvis, and adding to the difficulty. Finding the uterine contractions continuing with redoubled violence, and no progress being made, I was summoned in haste and found the patient in the condition above described.

It was with great difficulty that I succeeded in introducing the hand; but, after many fruitless efforts, the feet were brought down, and a fœtus extracted, in a semi-asphyxiated state, which I succeeded in resuscitating. The placenta soon came away and the woman was made comfortable. The child lived only about three weeks, but the arm was so much injured that I think this in a great measure contributed to its death.

Case II.—Dec. 15th, 1858. Tenah, aged about 28, was taken this afternoon in labor, with twins—the first was born naturally, but dead; the second presented the arm, which the midwife endeavored to extricate, but was foiled, and under these circumstances "sent for the doctor." After some trouble and delay, I succeeded in bringing down the feet and effecting delivery. Child dead. Placenta came away soon after.

Case III.—Sept. 3rd, 1859. Maria, aged about 25, was taken in labor, yesterday morning, with her first child. Labor progressed favorably until 4 o'clock this morning, when the membranes ruptured and head presented. The pains were very violent and quite expulsive in character, but were inadequate to effect anything, the head being locked in the inferior strait of the pelvis. In this condition I found the patient, when summoned to see her about 9 o'clock, A. M. On careful examination, I found a large tumor of the scalp from the continued pressure, and from this circumstance it was difficult to determine the particular presentation, but I concluded that the vertex was jammed behind the pubis and the forehead in front of the promontory of the sacrum.—With this view of the case I attempted the introduction of the forceps; the first blade was introduced without much trouble, the second occasioned me some embarrasment; but I finally succeeded in having both properly adjusted, and proceeded to extract the child, which I very readily accomplished. The placenta soon followed, and I left the patient comfortable. The child was barely alive, but survived thirty-six hours. The pressure upon the head and the subsequent extraction by the forceps had produced too much injury to hope for any other result. Sept. 4th, patient complains of soreness, &c., but otherwise doing well. She entirely recovered.

Case IV.—Sept. 8th, 1859. Lizzie, aged about 30, was taken in labor with twins, early in the evening, one of which was born early this morning. I was summoned about 11 o'clock, A. M., and found arm presenting. I had not much difficulty in reaching the feet in this case and effecting the delivery. Child alive; woman did well.

Case V.—Dec. 1st, 1859. Camilla, aged about 35, the mother of several children, has been in labor since yesterday. The waters have been discharged, and the pains are violent, but of no avail. The arm presents and is much tumefied. The uterine contractions were so great that I began to despair before making the attempt. Having ascertained from the presenting member the relation the fœtus bore to the maternal parts, I commenced my trials. After frequent ineffectual attempts to introduce the hand, I began to think of depleting measures to allay uterine contraction, but determined to make another effort, when I succeeded in reaching one of the feet; but my hand was so cramped that it was powerless, and I was much disappointed in being obliged to withdraw it. I was, however, encouraged in this attempt and determined to make another, which succeeded, and the version was effected. The subsequent steps of the operation were as in preceding cases. The child was, of course, dead.

I regret to say the sequelæ of this case were protracted and troublesome. A few days after her accouchment she complained of pain and soreness about the hypogastrium, extending down the thighs. The pulse was small and frequent, and I began to fear for the safety of my patient. On examination, per vaginam, I found the os uteri normal, and there was no tenderness to the touch; the tongue was also healthy. I had no reason to believe that her symptoms were those of metritis, although I dreaded such an occurrence. A mercurial plan of treatment was pursued, with occasional doses of an opiate, but the symptoms did not improve. The lochia was normal as to quality and quantity. Upon a closer examination, a few days after, I found her symptoms were of such a nature as to lead me to suppose that there was a pelvic abscess forming. My diagnosis was verified to the full extent. About the 1st of January, one month after her accouchment, while moving in bed, she felt something give way, and I was called to see her. A small orifice above the the pubis was discovered, from which the pus was oozing.—I enlarged the opening, and an immense quantity of pus was evacuated. The abscess had its origin in or about the pelvic fascia, had burrowed between the muscles and pointed as above detailed. My probe passed in almost every direction, but more particularly to the right side, and it was evident that the matter had burrowed beneath Poupart's ligament, as fluctuation was detected in the groin, where I made a counter-opening. Being the most dependent part of the abscess, this was afterwards the main orifice; the other soon healed. My patient was much weakened from this long and continued drain, but by the aid of tonics, a nutricious diet, and a good constitution, she entirely recovered.

Case VI.—May 16th, 1860. Was summoned to see Judy, who had been in labor since yesterday morning. About midnight, the membranes ruptured and arm presented. The details of this case are similar to the above. Several hours elapsed before I succeeded in effecting the delivery of a dead child. Patient did well.

These cases are interesting in many particulars. I have not given the sexes of the children, having neglected to make the observation during the trying and perplexing circumstances under which I was placed.

We have many pages written on the subject of shoulder and arm presentations, and the methods of procedure recommended are minute and every way admirable; but the student, however satisfied he may be with the instructions of his text-book and favorite authors, will find himself sadly puzzled when he is called upon for the first time to experience realities and apply his knowledge. After all, a sound judgment and thorough acquaintance with the general principles of his art must guide him to the special indications of each case. They also teach us the necessity of perseverence even under apparently the most hopeless circumstances, before we resort to extreme measures, and, perhaps, hazard the life of the unfortunate patient. *Nil desperandum. Labor vincit omnia.*—*Charleston Med. Journal.*

